# Kinesiophobia and Work Disability in Fibromyalgia: Cognitive Mediation in a Population-Based Study of Women

**DOI:** 10.3390/ejihpe16060072

**Published:** 2026-05-24

**Authors:** Giordano Mayer De Freitas, Guilherme Teixeira Lopes, Graziele Borges Bueno, Mariana Lentino Coelho, Julia Gomes, Caroline Leffa Venturini, Maria Eduarda Louzada, Sara Machado Peres, Barbara Regina França, Iraci L. S. Torres Pham, Felipe Fregni, Andrea Cristiane Janz Moreira, Wolnei Caumo

**Affiliations:** 1 Laboratory of Pain and Neuromodulation, Universidade Federal do Rio Grande do Sul (UFRGS), Porto Alegre 90035-003, Brazil; giordanofreitas_pes@hcpa.edu.br (G.M.D.F.); gtlopes@hcpa.edu.br (G.T.L.); gbbueno@hcpa.edu.br (G.B.B.); juliaggomes@hcpa.edu.br (J.G.); cventurini@hcpa.edu.br (C.L.V.); meloliveira@hcpa.edu.br (M.E.L.); iltorres@hcpa.edu.br (I.L.S.T.P.); ajmoreira@hcpa.edu.br (A.C.J.M.); 2 Post-Graduate Program in Medical Science, School of Medicine, Universidade Federal do Rio Grande do Sul (UFRGS), Porto Alegre 90035-003, Brazil; 3 Laboratório de Farmacologia da Dor e Neuromodulação, Investigações Pré Clínicas, Centro de Pesquisa Experimental, Hospital de Clínicas de Porto Alegre (HCPA), Porto Alegre 90035-003, Brazil; 4 Laboratory of Neuromodulation and Center for Clinical Research Learning, Physics and Rehabilitation Department, Spaulding Rehabilitation Hospital, Boston, MA 02114, USA; ffregni@post.harvard.edu; 5 Neuromodulation Center and Center for Clinical Research Learning, Spaulding Rehabilitation Hospital and Massachusetts General Hospital, Harvard Medical School, Boston, MA 02114, USA; 6 Pain and Palliative Care Service, Hospital de Clínicas de Porto Alegre (HCPA), Porto Alegre 90035-003, Brazil; 7 Surgery Department, School of Medicine, Universidade Federal do Rio Grande do Sul (UFRGS), Porto Alegre 90035-003, Brazil

**Keywords:** fibromyalgia, kinesiophobia, pain catastrophizing, work disability, rehabilitation, chronic pain

## Abstract

Background: Work disability in fibromyalgia is only partially explained by symptom severity, suggesting a relevant contribution of cognitive–behavioral mechanisms. Objective: This study aimed to determine whether kinesiophobia is associated with fibromyalgia impact and work-related disability and to assess whether pain catastrophizing mediates these relationships within a hierarchical biopsychosocial framework. Methods: This cross-sectional study included 2096 women with fibromyalgia recruited through a nationwide online survey. Participants completed validated instruments assessing fibromyalgia impact (FIQ), pain catastrophizing (PCS), depressive symptoms (PHQ-9), central sensitization (CSI), and kinesiophobia (Tampa Scale). Pain-related work disability was defined using the Graded Chronic Pain Scale–Revised (GCPS-R). Hierarchical logistic regression models identified factors independently associated with work disability. Mediation was tested using bootstrapped analyses (5000 resamples). Results: Kinesiophobia demonstrated a robust independent association with work disability (OR 1.03; 95% CI 1.02–1.05) after adjustment for sociodemographic factors, clinical pain phenotype, systemic burden, pain severity, psychocognitive load, and medication burden. Other relevant contributors included pain severity (OR 1.96; 95% CI 1.70–2.27), psychocognitive burden (OR 1.35; 95% CI 1.15–1.58), use of benzodiazepines (OR 1.74; 95% CI 1.33–2.28), and opioid use (OR 1.29; 95% CI 1.06–1.56). Mediation analysis indicated a significant indirect effect of kinesiophobia on work disability through pain catastrophizing (β = 0.131; 95% CI 0.078–0.188). Conclusions: Kinesiophobia is a proximal determinant of work disability in fibromyalgia, exerting direct and cognitively mediated effects through pain catastrophizing, reinforcing the fear-avoidance framework and the need for psychologically informed rehabilitation.

## 1. Introduction

Fibromyalgia (FM) is a chronic pain syndrome with an estimated prevalence of 2–5%, predominantly affecting women of working age (Gyorfi et al., 2022). FM is marked by widespread musculoskeletal pain, fatigue, sleep disturbances, cognitive dysfunction, and affective symptoms such as anxiety and depression, leading to substantial functional impairment and reduced quality of life (Clauw, 2015). Classified as a nociplastic pain condition, FM involves dysfunctions in central pain modulation, including nociceptive amplification, reduced descending inhibition, persistent central sensitization, and neuroinflammation (Treede et al., 2019; Findeisen et al., 2025), reflecting the interaction of biological, psychological, and social factors.

In Brazil, FM affects approximately 2.5% of the adult population, a prevalence similar to global estimates (Rosa Neto & de Carvalho, 2009). More broadly, chronic pain affects nearly 40% of Brazilian adults and represents one of the leading causes of disability, prolonged treatment, and early retirement among individuals of productive age (Breivik et al., 2013). The socioeconomic burden associated with chronic musculoskeletal pain is substantial and may exceed that observed in other chronic diseases, including diabetes, cardiovascular disorders, and cancer (Gaskin & Richard, 2012). In addition to direct healthcare costs, fibromyalgia is associated with major indirect costs related to unemployment and reduced work productivity, with approximately 25% of affected individuals becoming unable to maintain employment (Soroosh & Farbod, 2024). Recent international evidence further demonstrated increased healthcare expenditures, reduced income, and higher disability pension rates among patients with fibromyalgia (Amris et al., 2024), reinforcing the need to investigate mechanisms associated with functional disability in FM.

Among psychological factors linked to disability in chronic pain, kinesiophobia stands out. It is an excessive fear of movement, driven by concerns about pain or injury. Evidence shows kinesiophobia relates to reduced physical performance, altered proprioception, and less muscular endurance, regardless of pain intensity. It may limit rehabilitation by reinforcing threat perception and facilitating transition from persistent pain to disability (Saki et al., 2026; Goubran et al., 2025; Luque-Suarez et al., 2019). In chronic pain, kinesiophobia affects 50–70% of individuals and can stem from direct pain or social and observational learning (Schmid et al., 2021; Huang et al., 2022). In fibromyalgia, kinesiophobia often co-occurs with pain catastrophizing—a pattern marked by rumination, magnification, and helplessness (Sullivan et al., 2001). Together, these factors increase hypervigilance and avoidance, perpetuating a cycle of inactivity, pain, and limitation (Vlaeyen et al., 1995). These phenomena reflect neuroplastic processes underlying both adaptation and central sensitization (Hiraga et al., 2022; Palmer et al., 2023). Emotional symptoms and psychiatric diagnoses often coexist with chronic pain, but current evidence supports a cyclical, interdependent relationship rather than a direct causal one. Thus, kinesiophobia is a modifiable, clinically relevant factor (Martinez-Calderon et al., 2021).

Robust evidence shows that physical activity and therapeutic exercise promote adaptive neuroplasticity. They enhance descending pain modulation through somatosensory and neuroendocrine mechanisms (Palmer et al., 2023; He et al., 2024; Mansoor et al., 2025). International guidelines recommend physical exercise, combined with pain education and cognitive interventions, as a first-line non-pharmacological approach for fibromyalgia (Jones et al., 2024). Clinical trials and meta-analyses demonstrate that multimodal programs consistently improve pain, physical function, and quality of life (Bidonde et al., 2017; Andrade & Dominski, 2021; Rodríguez-Almagro et al., 2023). However, treatment responses remain heterogeneous. Biopsychosocial factors such as kinesiophobia, catastrophizing pain, depressive symptoms, and the degree of central sensitization strongly influence these outcomes. These factors may also amplify functional disability (Goubran et al., 2023).

Despite recognition of the importance of these factors, key gaps remain regarding their relative and interdependent contributions to functional outcomes, suggesting that systemic burden may increase vulnerability to maladaptive cognitive processing, which in turn influences behavioral avoidance and functional limitation. This study aimed to examine the association between kinesiophobia and clinical outcomes (fibromyalgia impact and work-related disability) within a hierarchical biopsychosocial framework and to evaluate whether pain catastrophizing mediates these relationships. To address this, hierarchical regression models were applied to account for demographic, clinical, and symptom-related factors; medication load and mediation analyses were conducted to explore the extent to which maladaptive pain-related cognitions may contribute to this association. Furthermore, we presumed that these cognitive–emotional mechanisms play a more central role in functional impairment than biological factors, such as pain severity or medication load.

## 2. Materials and Methods

### 2.1. Design Overview, Setting, and Participants

This cross-sectional study followed STROBE guidelines. All participants provided written informed consent. The Research Ethics Committee of Hospital de Clínicas de Porto Alegre (Institutional Review Board—IRB # 2023-0210) approved the protocol. The recruitment period for this study began on 1 August 2023, and ended on 30 November 2023.

### 2.2. Recruitment, Inclusion, and Exclusion Criteria

Individuals volunteered after responding to announcements in public spaces or advertisements on websites such as Facebook and Craigslist. These advertisements were shared through the networks of the National Association of Fibromyalgia and Related Diseases (ANFIBRO). A live online session introduced the project and addressed public questions. A message explaining the purpose of the study was shared on social media to invite people to take part.

Interested individuals accessed the study through a provided link. After confirming their interest, they received an informed consent form. Upon signing, participants received a questionnaire about sociodemographic data and the American College of Rheumatology (ACR-2016) questionnaire (Wolfe et al., 2016), via the Research Electronic Data Capture platform—REDCap. The evaluation collected medical history and symptom details to confirm diagnoses. Participants also completed a structured clinical screening that included symptom duration, widespread pain distribution, psychiatric history, chronic comorbidities, and current pharmacological treatment. Eligibility was confirmed only when all 2016 ACR diagnostic criteria for fibromyalgia were fulfilled. The research team managed recruitment by sending up to three messages per individual. If registration was still incomplete after ten days, an additional reminder was sent. After participants completed their registration, they did not receive any additional messages.

**Figure 1:**
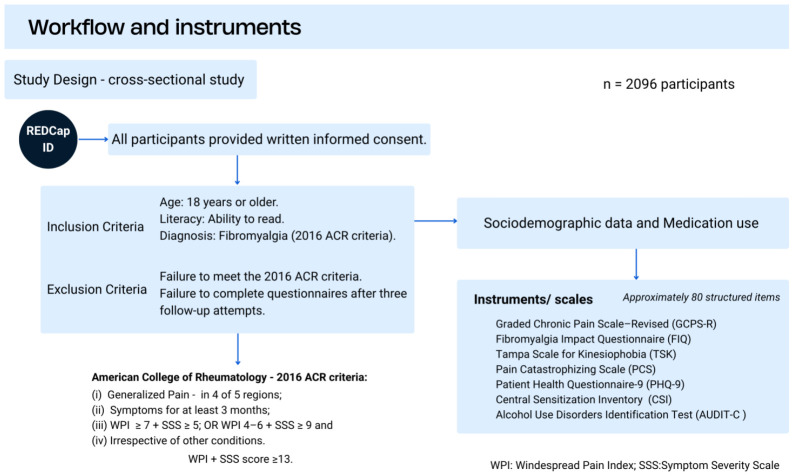
Participant assessment workflow and instruments (*n* = 2096 participants).

To participate, individuals had to be at least 18 years old, able to read, and have a confirmed fibromyalgia diagnosis according to the 2016 criteria set by the American College of Rheumatology (Wolfe et al., 2016). The 2016 ACR criteria stipulate that: (i) generalized pain, defined as pain in at least 4 of 5 regions, must be present; (ii) symptoms must be persistent for at least 3 months; (iii) the Widespread Pain Index (WPI) must be ≥7 with a Symptom Severity Scale (SSS) score ≥ 5, or a WPI of 4–6 with an SSS score ≥ 9; and (iv) the diagnosis is valid regardless of other comorbid conditions, and the presence of fibromyalgia does not exclude additional clinically relevant illnesses. Consistent with these criteria, participants needed a combined WPI + SSS score ≥ 13. Participants were excluded if they did not meet the 2016 ACR criteria or did not complete study questionnaires after three follow-up attempts (Figure 1).

### 2.3. Instruments and Assessment

After providing consent, participants completed the questionnaires via REDCap. To minimize misunderstanding, each item included accessible instructions. All instruments were validated for the Brazilian population, ensuring accurate and reliable measurement. Overall, the multidimensional assessment comprised approximately 80 structured items covering pain severity, disability, fibromyalgia impact, cognition, mood, central sensitization, medication use, and lifestyle-related domains. We emphasize, however, that all measures were self-reported. The research team was trained to guide the recruitment process and instruct participants on how to respond to the study demands. Response monitoring was conducted continuously, and in cases of incomplete answers, up to three follow-up contacts were made to ensure correction. Weekly meetings were held to discuss any doubts that arose during the study, enabling evaluators to calibrate. Instructions for all procedures and assessment methods were included in a standardized instruction manual.

#### 2.3.1. Primary and Secondary Outcomes

Primary and secondary outcomes: the primary outcome was the pain that limited daily activities or work on most days for at least three months. The secondary outcome was the impact of fibromyalgia symptoms on quality of life assessed by the Fibromyalgia Impact Questionnaire (FIQ) (Marques et al., 2006).

#### 2.3.2. Outcomes Measures (Dependent Variable)

(a) The dependent variable was pain-related work disability, operationalized as a dichotomous outcome. Pain-related work disability was assessed using the Graded Chronic Pain Scale–Revised (GCPS-R), with item 6 specifically evaluating pain-related work limitations. Participants classified as grade 3 (high-impact chronic pain)—defined as pain that limited daily activities or work on most days for at least three months—were categorized as incapacitated due to pain, whereas those classified as grades 0–2 were categorized as not incapacitated. This binary variable was used as the dependent outcome in the logistic regression and mediation analyses.
(b) The Fibromyalgia Impact Questionnaire (FIQ) was used to assess the impact of fibromyalgia on function, symptoms, and overall quality of life across 10 domains with a score ranging from 0 to 100, with higher scores indicating greater disease burden (e.g., physical functioning, pain intensity, fatigue, stiffness, sleep quality, mood, work impairment, and ability to perform daily activities). We used the version validated for the Brazilian population (Marques et al., 2006).

#### 2.3.3. Covariate Measures

(c) Kinesiophobia is assessed using the Tampa Scale for Kinesiophobia (TSK), a self-report measure of fear of movement and re-injury. The original version comprises 17 items rated on a 4-point Likert scale (1 = strongly disagree to 4 = strongly agree), yielding a total score ranging from 17 to 68, with higher scores indicating greater kinesiophobia. The TSK assesses maladaptive beliefs related to physical activity and injury vulnerability and has demonstrated good psychometric properties across chronic pain populations, including fibromyalgia. In this study, the total TSK score was analyzed as a continuous variable. It is the main interest factor (Siqueira et al., 2007).
(d) The Symptom Severity Scale (SSS) was used to assess the severity of three core somatic symptom domains—fatigue, symptoms of cognitive dysfunction and waking unrefreshed. We used the three corresponding items from the Symptom Severity Scale (SSS) of the 2016 revisions of the American College of Rheumatology (ACR) fibromyalgia diagnostic criteria (Wolfe et al., 2016). Each symptom was rated according to its severity over the past week using a 0–3 scale: 0 = no problems; 1 = slight or mild problems, generally intermittent; 2 = moderate or considerable problems, often present and/or of moderate intensity; and 3 = severe, pervasive, continuous, or life-disturbing problems.
(e) The Pain Catastrophizing Scale (PCS) was used to assess catastrophic thinking related to pain. It contains 13 items scored 0–4 (total 0–52) covering rumination, magnification, and helplessness domains (Caumo et al., 2013).
(f) The Patient Health Questionnaire-9 (PHQ-9) was used to evaluate depressive symptoms over the past 15 days. The scale includes nine DSM (Diagnostic and Statistical Manual of Mental Disorders) based on 9 items scored 0–27; in Brazil, a cutoff ≥ 9 indicates clinically relevant depression (Santos et al., 2013).
(g) The Central Sensitization Inventory—Brazilian Version (CSI) was used to screen for central sensitization symptoms. This 25-item scale (0–100) evaluates somatic and emotional symptoms commonly associated with central sensitization, including diffuse pain, fatigue, non-restorative sleep, cognitive difficulties, headaches, and urological complaints (Caumo et al., 2017).
(h) AUDIT-C was used to assess alcohol consumption patterns and identify risk related to alcohol use. It consists of three items scored 0–4 (Corradi-Webster et al., 2005).
(i) Sociodemographic and clinical covariates: sociodemographic and clinical covariates were collected using a standardized questionnaire and included age (years), educational attainment (years or highest level of formal schooling), body mass index (BMI) category, occupational status (unemployed; employed/student/self-employed; retired; receiving disability benefits), and fibromyalgia duration (years).
(j) Medication use was categorized a priori into the following groups: (i) Antidepressants, including tricyclic antidepressants, dual-action antidepressants, selective serotonin reuptake inhibitors (SSRIs), and a combined tricyclic/dual-action indicator; (ii) Sleep-related medications, including melatonin, zolpidem, and benzodiazepines; and (iii) Analgesics, including non-opioid analgesics (nonsteroidal anti-inflammatory drugs, dipyrone, and acetaminophen); any opioid use (binary indicator); and specific opioid agents (codeine, morphine, oxycodone, methadone, and tramadol).

### 2.4. Efforts to Address Potential Sources of Bias

To minimize potential sources of bias, all study procedures were fully standardized through REDCap, ensuring identical administration, automated data capture, and removal of duplicate entries. Recruitment was broad and public, and all instruments used were validated for the 2096 participants, reducing measurement error. Sensitive items (e.g., trauma exposures) were self-administered online to limit bias to social desirability. The research team followed a structured operations manual, with weekly calibration meetings and continuous data-quality monitoring. Incomplete responses triggered up to three automated reminders to reduce attrition bias.

### 2.5. Sample Size Calculation

The sample estimate was based on data from a latent class model applied to a research sample conducted by our group in the state of Rio Grande do Sul. This model identifies ordinal classes that represent levels of severity related to the impact of fibromyalgia symptoms on quality of life. The prevalence of cases classified in the high severity class was considered the primary outcome. The calculation aimed to estimate the proportion of occurrence of fibromyalgia with high severity, adopting a confidence level of 95% and a total amplitude of the confidence interval of 2% (margin of error of ±1%). The calculation was made based on the 1:3 ratio of occurrence of the disability outcome (*n* = 2090) and we added to the calculation losses and refusals, which were estimated at 25%, totaling a sample *n* of 2600 participants with fibromyalgia. The total valid *n* for the study was 2096.

### 2.6. Statistical Analysis

Participants were stratified according to work disability status. Continuous variables were summarized as mean and standard deviation (SD) or median and interquartile range (IQR), according to data distribution, while categorical variables were expressed as absolute and relative frequencies. Normality was assessed using the Shapiro–Wilk and Kolmogorov–Smirnov tests.

Group comparisons were performed using χ^2^ tests for categorical variables and independent t-tests for continuous variables, as appropriate. To estimate independent associations with work disability, binary logistic regression models were performed. Variables associated with the outcome at *p* < 0.20 in univariable analyses, as well as those considered clinically relevant, were considered eligible for multivariable modeling. Multivariable analyses followed a hierarchical conceptual modeling strategy based on a biopsychosocial framework, in which variables were entered according to their theoretical proximity to the outcome. Distal variables included sociodemographic factors (age and education). Intermediate variables comprised clinical and treatment-related domains operationalized through composite indices, including systemic burden, pain severity, psychocognitive burden, psychopharmacological load, and analgesic load. Proximal variables reflected cognitive–behavioral mechanisms, specifically pain catastrophizing and kinesiophobia.

Composite indices were constructed a priori to reduce dimensionality, minimize multicollinearity among highly correlated clinical variables, and improve interpretability within the hierarchical biopsychosocial model. Each index represented a theoretically coherent clinical domain rather than isolated predictors. The systemic burden index integrated cumulative abuse exposure, central sensitization symptoms, and the number of chronic diseases. The pain severity index summarized widespread pain severity, recent pain intensity, and pain interference. The psychocognitive burden index combined depressive symptoms, cognitive symptoms, and sleep-related impairments. Medication-related indices represented cumulative psychopharmacological and analgesic burden. When variables were measured on different scales, standardized z-scores were used before averaging. Variables were entered sequentially according to hierarchical levels (Rothman et al., 2008). Variables retained at each level remained in subsequent models regardless of statistical significance, preserving theoretical consistency and avoiding inappropriate adjustment for potential mediators.

Odds ratios (ORs) were interpreted according to the stage at which variables were first introduced, minimizing attenuation of effects due to overadjustment (Zeng et al., 2021). In the first stage, distal demographic variables were entered (Caumo et al., 2001). Intermediate composite indices were included in the second stage, followed by proximal cognitive–behavioral variables in the final stage. Within each hierarchical block, variables were entered using a forward stepwise procedure (Heinze et al., 2018). Variable retention was guided not only by statistical significance (*p* < 0.05) but also by conceptual relevance, stability of effect estimates (Mickey & Greenland, 1989), and contribution to overall model fit (Hosmer et al., 2013). Model comparisons and hypothesis testing were based on likelihood ratio tests. Multicollinearity was assessed using variance inflation factors (VIF) and tolerance statistics (Senaviratna & Cooray, 2019). Overall model adequacy was evaluated through calibration and discrimination analyses using the Hosmer–Lemeshow goodness-of-fit test and the area under the receiver operating characteristic curve (AUC/ROC), respectively. Results are presented as odds ratios (ORs), 95% confidence intervals (95% CIs), and *p*-values.

Mediation analyses were performed to evaluate whether pain catastrophizing mediated the association between kinesiophobia and work disability. Indirect effects were estimated using bootstrapped confidence intervals based on 5000 resamples. Statistical significance was defined as *p* < 0.05.

## 3. Results

### 3.1. Sample Characteristics and Work Disability Status

From the publicly available online survey widely disseminated across national platforms, 4200 individuals initiated the questionnaire. After identifying and removing repeated attempts by the same participant, 3500 unique respondents remained. Among these, 2498 (71.4%) completed all required instruments. A total of 301 respondents did not meet the fibromyalgia screening criteria and were excluded, yielding 2197 eligible cases. Because only 101 men completed the full survey—too few for trustworthy analyses separating results by sex—these participants were excluded from analysis. The final analytical cohort therefore comprised 2096 women, representing 49.9% of all individuals who initiated the survey and 83.9% of those who completed all questionnaires.

Table 1 summarizes the sociodemographic and clinical characteristics, comorbidities, medication use, and health care engagement indicators of the study sample, stratified by pain-related work disability. Overall, participants were predominantly middle-aged women with long-standing fibromyalgia, high rates of medical comorbidities, and extensive use of psychotropic and analgesic medications, reflecting a population with substantial clinical complexity.

### 3.2. Clinical, Psychological, and Functional Differences According to Work Disability

Table 2 displays fibromyalgia severity, pain interference, psychological variables, central sensitization, psychiatric comorbidities, adverse life events, and functional outcomes according to pain-related work disability. Participants with work disability showed consistently higher symptom burden and worse psychological and functional profiles.

### 3.3. Hierarchical Logistic Regression: Factors Independently Associated with Work Disability

A hierarchical logistic regression model was constructed to evaluate factors associated with pain-related disability (Table 3). Sociodemographic variables were entered first, followed by systemic burden, pain severity, psychocognitive burden, medication load, and finally kinesiophobia, reflecting increasing conceptual proximity to disability. OR are presented at the step in which each variable entered the hierarchical model, allowing assessment of their independent contribution prior to adjustment for more proximal factors. Distal variables showed modest associations with disability. Systemic burden and pain severity were associated with disability at entry but were attenuated after inclusion of psychocognitive factors. Notably, kinesiophobia (TSK) entered at the final stage and demonstrated a robust independent association, supporting its role as a proximal determinant of disability.

Interpretation: Effects are shown at the point of entry in the hierarchical model, allowing visualization of attenuation or persistence across blocks. Kinesiophobia remains a proximal determinant of disability.

### 3.4. Cognitive Mediation of the Relationship Between Kinesiophobia, Work Disability, and Disease Impact on Quality of Life

**Figure 2:**
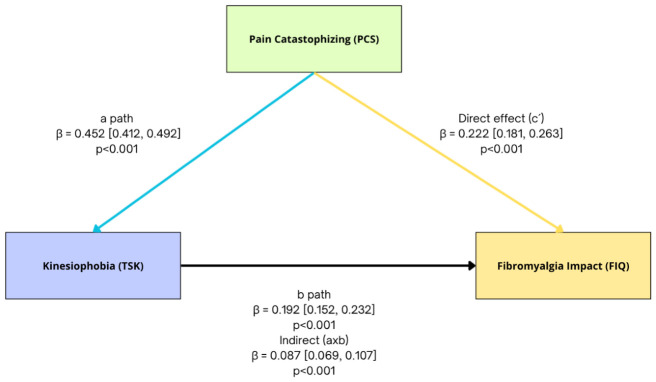
Mediation model examining the association between pain catastrophizing and fibromyalgia impact on quality of life, as assessed by the Fibromyalgia Impact Questionnaire, through kinesiophobia. Path *a* represents the association between pain catastrophizing and kinesiophobia. Path *b* represents the association between kinesiophobia and fibromyalgia impact. Path *c’* represents the direct effect of pain catastrophizing on fibromyalgia impact after accounting for the mediator. The indirect effect *(a × b)* represents the mediated pathway through kinesiophobia. Values displayed in the figure correspond to regression coefficients (β or OR), 95% confidence intervals (CI), and *p*-values derived from the fully adjusted model. *TSK: Tampa Scale of Kinesiophobia; PCS Pain Catastrophizing Scale; FIQ: Fibromyalgia Impact Questionnaire*.

Figure 2 illustrates a partial mediation model in which kinesiophobia significantly mediates the association between pain catastrophizing and fibromyalgia impact (FIQ), with a remaining direct effect after adjustment for age, education, number of non-opioid analgesics, number of opioid analgesics, and medication burden (indices comprised the sum of anticonvulsants, any antidepressants, benzodiazepines, and zolpidem).

**Figure 3:**
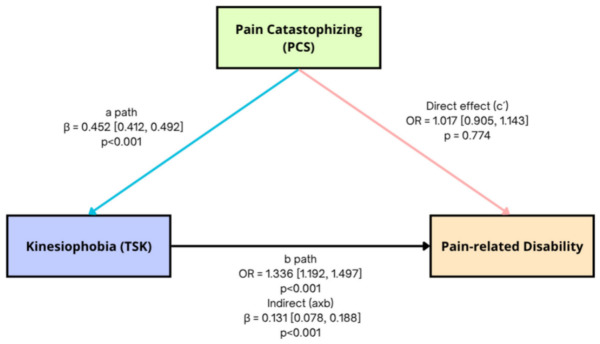
Mediation model examining the association between pain catastrophizing and pain-related disability through kinesiophobia. Path *a* represents the association between pain catastrophizing and kinesiophobia. Path *b* represents the association between kinesiophobia and pain-related disability. Path *c’* represents the direct effect of pain catastrophizing on pain-related disability after accounting for the mediator. The indirect effect *(a × b)* represents the mediated pathway through kinesiophobia. Values displayed in the figure correspond to regression coefficients (β or OR), 95% confidence intervals (CI), and *p*-values derived from the fully adjusted model. *TSK: Tampa Scale of Kinesiophobia; PCS Pain Catastrophizing Scale*.

Figure 3 depicts a mediation model in which kinesiophobia fully mediates the association between pain catastrophizing and pain-related disability, as the direct effect is no longer significant after adjustment while the indirect effect remains significant. All estimates are adjusted for age, education, number of non-opioid analgesics, number of opioid analgesics, and medication burden (indices comprised the sum of anticonvulsants, any antidepressants, benzodiazepines, and zolpidem).

## 4. Discussion

This study provides novel evidence that pain catastrophizing functions as a central cognitive mediator linking kinesiophobia to clinically relevant outcomes in fibromyalgia within a hierarchical biopsychosocial framework that integrates overall pain burden and pharmacological load. While prior models have typically conceptualized catastrophizing as an antecedent of fear-avoidance, our findings support an alternative pathway in which fear of movement is associated with maladaptive cognitive processing, which in turn is linked to functional impairment. Consistent with this framework, kinesiophobia was significantly associated with pain catastrophizing, which in turn was associated with both fibromyalgia impact and work disability. Mediation analyses demonstrated that pain catastrophizing partially mediated the association between kinesiophobia and fibromyalgia impact and fully mediated its association with work disability. These associations remained robust after adjustment for sociodemographic factors, pain severity, and pharmacological burden, suggesting that cognitive processing of pain represents a key mechanism through which behavioral fear responses translate into functional impairment. This pattern aligns with the foundational fear-avoidance model proposed by Vlaeyen and Linton (Vlaeyen & Linton, 2000), in which fear-driven interpretations of pain escalate avoidance behaviors and amplify disability. These multivariable models further revealed that kinesiophobia emerged as an independent proximal determinant of work disability, even after accounting for systemic burden, psychocognitive factors, and medication use, reinforcing its central role in disability pathways. This supports the conceptual shift that cognitive mechanisms—especially catastrophic thinking—should be prioritized as therapeutic targets in individuals presenting with high movement-related fear.

Mediation analysis demonstrated a significant indirect effect of kinesiophobia on work disability through pain catastrophizing (β = 0.131; 95% CI 0.078–0.188), supporting the hypothesis that maladaptive cognitive processing partially explains the relationship between fear of movement and functional impairment. Although the magnitude of the association between kinesiophobia and work disability was modest (OR 1.03; 95% CI 1.02–1.05), this effect remained independent after adjustment for pain severity, systemic burden, psychocognitive burden, and medication load, reinforcing the clinical relevance of fear-related behavioral responses within a multidimensional biopsychosocial framework. Previous studies in fibromyalgia and chronic pain populations have similarly demonstrated small-to-moderate associations between kinesiophobia, disability, and reduced quality of life, with correlation coefficients ranging from r = 0.26 to 0.55 (Zale et al., 2013). In occupational chronic low back pain populations, kinesiophobia and fear-avoidance beliefs were associated with disability and prolonged work absence (r = 0.26–0.34) (Macías-Toronjo et al., 2020). Likewise, exposure-based interventions targeting fear-related avoidance behaviors have demonstrated large improvements in behavioral functioning (d = 1.63–2.31), reinforcing the role of maladaptive fear processing in sustaining disability (Flink et al., 2013).

These behavioral findings are biologically plausible and consistent with neuroimaging evidence demonstrating altered functional connectivity in neural networks involved in salience attribution, affective regulation, and cognitive control in fibromyalgia. Resting-state neuroimaging studies involving 1877 individuals (947 patients and 930 controls) demonstrated increased functional connectivity between the dorsolateral prefrontal cortex (DLPFC) and anterior cingulate cortex (ACC), whereas reduced insula–ACC connectivity was associated with greater pain intensity, catastrophizing, and depressive symptom severity (r = −0.58 to −0.34) (Tocchetto et al., 2025). These alterations have been linked to impaired affective pain regulation, heightened salience attribution, emotional distress, cognitive dysfunction, and reduced executive regulation mediated by fronto-cingulate networks (Schlösser et al., 2008). Consistent with these findings, previous studies have shown that catastrophizing is independently associated with greater pain intensity, psychological distress, pain interference, poorer physical functioning, and disability in chronic pain and fibromyalgia populations. Catastrophizing alone explained an additional 29% of the variance in pain intensity, 30% of psychological distress, and 11% of pain-related disability after adjustment for demographic and clinical factors, while correlations with pain interference and physical functioning ranged from r = 0.53 to 0.70 and r = −0.39 to −0.67, respectively (McDermid et al., 1996; Borg et al., 2018). Additional resting-state fMRI studies further demonstrated increased connectivity between the default mode network and insular regions in fibromyalgia, with stronger insula–network coupling associated with higher spontaneous pain intensity during scanning (Napadow et al., 2010). Because these neural networks are critically involved in salience processing and top-down modulation of pain, such findings support the hypothesis that maladaptive cognitive–emotional responses—including catastrophizing and kinesiophobia—may contribute to hypervigilance, amplified threat perception, and functional impairment in fibromyalgia.

Clinically, these findings suggest that kinesiophobia should not be viewed as an isolated therapeutic target but rather as part of a broader cognitive–emotional process associated with functional impairment in fibromyalgia. Within this framework, fear of movement interacts with maladaptive pain-related cognitions, particularly catastrophizing, contributing to avoidance behaviors, disability, and poorer treatment response. Accordingly, interventions focused exclusively on reducing fear of movement may have limited effectiveness if catastrophic interpretations of pain are not simultaneously addressed. This interpretation is consistent with evidence showing that psychological interventions tend to achieve greater clinical benefit when behavioral and cognitive components are addressed concurrently. Exposure-based and graded-activity approaches appear to be more effective when combined with strategies targeting catastrophic thinking and maladaptive pain beliefs. Supporting this perspective, a large network meta-analysis including 224 trials and more than 29,000 participants demonstrated that multimodal interventions integrating exercise, psychological therapies, and pain education produced the most consistent improvements in pain and physical function in fibromyalgia, with exercise-based approaches showing moderate-certainty evidence for clinically meaningful reductions in pain (SMD = −0.44 to −0.63) and improvements in physical functioning (SMD = −0.42 to −0.56) compared with usual care or non-exercise interventions (Ho et al., 2022). Together, these findings reinforce the relevance of multimodal approaches integrating cognitive–behavioral strategies, pain neuroscience education, and graded exposure as potentially more effective pathways for modifying disability trajectories in fibromyalgia. Overall, the present findings support the importance of psychologically informed pain management strategies in fibromyalgia care.

These findings also demonstrated that work disability and FIQ scores remained independently associated even after multivariable adjustment, supporting the interpretation that the FIQ reflects a broad multidimensional burden encompassing physical function, fatigue, pain severity, and emotional distress. While work disability captures a major functional consequence of fibromyalgia, previous studies have similarly shown that FIQ scores strongly correlate with participation restriction, psychosocial impairment, symptom severity, and overall disease impact. In multicomponent rehabilitation models, reductions greater than 14 points in pain catastrophizing and 11.4 points in kinesiophobia were associated with significant improvements in FIQ scores, reinforcing its value as a comprehensive indicator of fibromyalgia severity and functional status (Araya-Quintanilla et al., 2026). Mediation analyses further demonstrated that kinesiophobia exerted both direct and indirect effects on FIQ through pain catastrophizing, suggesting that fear of movement contributes not only to work-related disability but also to broader deterioration in quality of life and disease burden. Together, these findings support the interpretation that disability in fibromyalgia is embedded within a multidimensional clinical context rather than being explained by isolated symptoms alone.

The systemic burden index, which integrated cumulative abuse exposure, central sensitization-related symptoms, and chronic comorbidity load, remained independently associated with work disability, although its magnitude was attenuated after inclusion of cognitive–emotional variables. This finding suggests that disability in fibromyalgia emerges within a broader biopsychosocial context rather than from isolated symptom domains alone. Because central sensitization was incorporated as part of a multidimensional systemic burden construct, rather than analyzed as an isolated predictor, its contribution should be interpreted as reflecting a broader pattern of multisystem symptom amplification interacting with cumulative stress exposure and medical comorbidity. Notably, after inclusion of proximal cognitive–emotional variables, the explanatory contribution of systemic burden decreased, whereas kinesiophobia and pain catastrophizing demonstrated stronger associations with functional outcomes. These findings suggest that systemic biopsychosocial burden may represent a background vulnerability, while maladaptive cognitive processing more directly influences the extent of functional impairment. This interpretation is consistent with previous biopsychosocial models in fibromyalgia showing that depressive symptoms, pain catastrophizing, fatigue, sensitization-related symptoms, and pain severity interact to predict poorer physical functioning and reduced quality of life, with multidimensional models explaining approximately 34–53% of the variance in functional outcomes (Caumo et al., 2026). Current nociplastic pain models further support the concept that altered central nervous system processing contributes not only to pain amplification but also to persistent disability, fatigue, cognitive dysfunction, and reduced physical functioning in fibromyalgia (Fitzcharles et al., 2021). Together, these findings support a hierarchical interpretation in which systemic burden contributes to the clinical environment underlying symptom expression, whereas cognitive–emotional mechanisms—particularly kinesiophobia and catastrophizing—represent more proximal correlates of functional disability.

Work disability was independently associated with the use of anticonvulsants, benzodiazepines, and opioids; however, these associations should not be interpreted as causal. Rather, medication use likely reflects greater clinical complexity, symptom severity, and treatment refractoriness. In addition, pharmacological burden itself may contribute to reduced functional performance through mechanisms such as sedation, cognitive impairment, psychomotor slowing, and reduced alertness. Benzodiazepine use, in particular, may also reflect the presence of anxiety disorders, sleep disturbances, emotional distress, and heightened affective dysregulation, all of which are commonly associated with greater symptom severity and maladaptive pain-related cognitions in chronic pain populations. Supporting this interpretation, Cunningham et al. demonstrated that patients using benzodiazepines presented significantly greater depressive symptoms, pain catastrophizing, and pain severity scores compared with non-users, with depressive symptoms remaining independently associated with benzodiazepine use after multivariable adjustment (Cunningham et al., 2017). Previous studies in chronic pain populations have also shown that persistent opioid use and higher pharmacological burden are associated with greater psychological distress, pain catastrophizing, fear-avoidance behaviors, and poorer functional outcomes. In chronic non-cancer pain populations, Fear-Avoidance Beliefs Questionnaire–Physical Activity (FABQ-PA) scores were among the strongest predictors of unsuccessful opioid discontinuation (β = 7.27; 95% CI 5.43–9.11), while pain catastrophizing remained independently associated with persistent opioid reliance (β = 0.80; 95% CI 0.62–0.99) (Silva et al., 2021). In the present study, the persistence of medication-related associations after multivariable adjustment suggests that pharmacological burden may represent not only greater symptom severity but also a clinical profile characterized by higher psychological vulnerability and functional impairment.

To our knowledge, this study represents one of the largest population-based investigations conducted in South America, examining kinesiophobia and work disability in women with fibromyalgia within a hierarchical biopsychosocial framework. The hierarchical modeling approach allowed a structured evaluation of how biological, clinical, systemic, pharmacological, and cognitive–emotional domains interact in relation to functional disability. While the fear-avoidance model has been extensively investigated internationally, comparatively fewer large population-based studies have explored these associations in South American populations. Our findings suggest that cognitive–emotional mechanisms, particularly kinesiophobia and pain catastrophizing, remained independently associated with work disability even after adjustment for pain severity, systemic burden, and medication load. Rather than minimizing the importance of nociplastic pain severity, these findings support the interpretation that the cognitive and behavioral response to pain may substantially influence the extent of functional impairment. Overall, the present results reinforce the relevance of psychologically informed and multimodal rehabilitation strategies targeting maladaptive pain-related beliefs and behaviors within the broader biopsychosocial management of fibromyalgia.

## 5. Limitations

Several methodological considerations should be considered when interpreting these findings. *First*, the cross-sectional design precludes causal inference. Although mediation analyses were conducted within a theoretically guided hierarchical framework, they represent statistical mediation rather than temporal or mechanistic causation and therefore should be interpreted as hypothesis-generating rather than causal. *Second*, all measures were self-reported, which may introduce recall bias, mood-congruent reporting, and symptom amplification—particularly relevant in conditions characterized by cognitive load, affective dysregulation, and nociplastic pain. Although fibromyalgia eligibility was operationalized using the validated ACR-2016 criteria and structured clinical screening, direct face-to-face diagnostic confirmation by the research team was not feasible due to the nationwide online design. Work disability status was also based on self-report of being on work leave or receiving disability-related benefits, without differentiation between specific categories of benefits or assessment of socioeconomic and behavioral factors potentially associated with work-disability status. Therefore, the findings should not be interpreted as supporting causal assumptions regarding reinforcement mechanisms or “learned behavior” related to benefit receipt. Nevertheless, the instruments used are validated, widely applied in fibromyalgia research, and capture clinically meaningful subjective experiences that are integral to the disorder. *Third*, the study relied on composite indices (systemic burden, pain severity, psychocognitive burden, and medication load), which were constructed a priori to reduce dimensionality, avoid multicollinearity, and reflect theoretically coherent clinical domains. While this approach enhances model stability and interpretability, it may obscure the contribution of individual components, particularly central sensitization or cumulative trauma, which are embedded within broader indices rather than examined as isolated predictors. The hierarchical modeling approach, combined with assessment of collinearity (VIF and tolerance), minimized redundancy, but residual overlap among symptom-based constructs is unavoidable in fibromyalgia. *Fourth*, the exclusive inclusion of women limits generalizability to men, who represented a small proportion of the initial sample. Online recruitment may also have introduced selection bias toward individuals with higher symptom burden, greater digital engagement, or stronger illness identification. However, the large national sample, internal consistency of indices, and robustness of multivariable effects across hierarchical blocks mitigate concerns regarding sampling heterogeneity and enhance the credibility of the observed associations. Finally, cognitive–emotional factors such as catastrophizing and kinesiophobia, which emerged as strong proximal determinants, may not only act as mechanisms linking symptoms to disability but may also influence self-report accuracy, threat appraisal, and interpretation of symptom severity. This dual role—mechanistic and perceptual—should be acknowledged, and future longitudinal or experimental studies incorporating behavioral and neurophysiological measures are needed to clarify directionality.

## 6. Conclusions

These findings show that kinesiophobia is a proximal determinant of work disability in fibromyalgia, with part of its effect mediated by pain catastrophizing. Cognitive–emotional mechanisms therefore play a central role in linking nociplastic pain to functional impairment, surpassing the contributions of pain severity, systemic burden, and medication load. Together, these results support the fear-avoidance framework and highlight the need for rehabilitation strategies that address maladaptive beliefs and promote adaptive engagement in activity.

## Figures and Tables

**Table 1:** Sociodemographic and clinical characteristics, comorbidities, medication use, and health care engagement, stratified by pain-related work disability (*n* = 2096).

	Disability to Work		
Variable	No (*n* = 1036)	Yes (*n* = 1060)	OR (95% CI)
Age (yrs)	50.09 (±10.98)	51.28 (±9.39)	1.01 (1.00–1.02)
Formal education (yrs)	14.58 (±5.38)	13.17 (±5.46)	0.95 (0.94–0.97)
Alcohol Use Disorders Identification Test Consume (AUDIT-C) (yes)	640 (61.8)	504 (47.5)	0.56 (0.48–0.66)
Race Black/Brown (yes)	184 (17.8)	190 (17.9)	1.01 (0.80–1.27)
Smoke (yes)	138 (43.0)	183 (57.0)	1.61 (1.25–2.07)
Professional status *n* (%)			
Unemployed (yes)	85 (29.4)	204 (70.6)	3.01 (2.29–3.95)
Employed/Student/etc. (yes)	733 (64.9)	396 (35.1)	0.33 (0.28–0.39)
Retired (yes)	180 (49.7)	182 (50.3)	1.02 (0.80–1.29)
Disability benefits (yes)	38 (12.0)	278 (88.0)	9.60 (6.66–13.85)
Body mass index (BMI) *n* (%)			
Normal weight (BMI < 18.5–24.9 kg/m^2^) (yes)	253 (54.9)	208 (45.1)	0.78 (0.63–0.97)
Overweight (BMI 25.0–29.9 kg/m^2^) (yes)	366 (49.7)	371 (50.3)	1.03 (0.85–1.25)
Obesity (BMI ≥ 30 kg/m^2^) (yes)	411 (46.4)	475 (53.6)	1.33 (1.10–1.61)
Chronic disease *n* (%)			
Hypertension (HAS) (yes)	615 (44.3)	773 (55.7)	1.58 (1.34–1.86)
Diabetes (yes)	287 (46.1)	336 (53.9)	1.37 (1.12–1.68)
Stroke (yes)	99 (45.4)	119 (54.6)	1.20 (0.88–1.64)
Asthma (yes)	9 (33.3)	18 (66.7)	2.00 (0.88–4.52)
Chronic Obstructive Pulmonary Disease (COPD) (yes)	172 (39.0)	269 (61.0)	1.73 (1.39–2.16)
Number chronic diseases	1.14 (1.14)	1.42 (1.14)	1.22 (1.14–1.30)
Psychotropic medication use *n* (%)			
Use of anticonvulsants (yes)	501 (43.5)	652 (56.5)	1.58 (1.34–1.86)
Use of tricyclic antidepressants (yes)	244 (42.7)	327 (57.3)	1.57 (1.28–1.93)
Use of dual antidepressants (yes)	414 (47.8)	452 (52.2)	1.16 (0.98–1.38)
Use of benzodiazepines (yes)	101 (30.5)	230 (69.5)	3.04 (2.36–3.91)
Use of zolpidem (yes)	74 (41.1)	106 (58.9)	1.46 (1.05–2.03)
Use of carbamazepine (yes)	21 (42.9)	28 (57.1)	1.33 (0.72–2.45)
Use of gabapentin (yes)	490 (43.2)	645 (56.8)	1.60 (1.36–1.89)
Use of selective serotonin reuptake inhibitors (SSRI) (yes)	170 (41.5)	240 (58.5)	1.49 (1.18–1.87)
Use of melatonin (yes)	73 (45.1)	89 (54.9)	1.20 (0.86–1.68)
Opioid Analgesic use *n* (%)			
Non-opioid ≥ 2	737 (47.4)	819 (52.6)	1.23 (1.05–1.45)
Opioid use	648 (62.6)	985 (92.9)	7.80 (5.90–10.30)
Codeine	272 (42.0)	376 (58.0)	1.55 (1.28–1.87)
Tramadol	331 (40.8)	481 (59.2)	1.68 (1.40–2.02)
Morphine	29 (27.4)	77 (72.6)	3.10 (1.95–4.93)
Oxycodone	10 (29.4)	24 (70.6)	2.70 (1.22–5.98)
Methadone	6 (18.2)	27 (81.8)	5.15 (2.04–12.99)
Number of opioids analgesics	0.60 (±0.76)	0.86 (±0.83)	
Number of non-opioid analgesics	1.14 (±0.94)	1.28 (±0.95)	
Physical activity	532 (50.3)	526 (49.7)	0.97 (0.83–1.14)

Data were presented as mean (SD) or *n* (%), odds ratio (OR) with confidence interval (CI 95%).

**Table 2:** Clinical, psychological, and functional characteristics stratified by pain-related work disability. Data were presented as mean (SD) or *n* (%), odds ratio with confidence interval (CI 95%) (*n* = 2096).

	Disability to Work		
Variable	No (*n* = 1036)	Yes (*n* = 1060)	OR (95% CI)
Fibromyalgia—Symptom Severity and Diagnostic Criteria (ACR 2016)			
Widespread pain index (WPI)	10.28 (±3.19)	11.12 (±3.32)	1.08 (1.06–1.11)
Symptom Severity Score (SSS)	9.46 (±1.66)	10.09 (±1.42)	1.22 (1.16–1.29)
Fatigue	2.55 (±0.55)	2.76 (±0.46)	1.85 (1.60–2.14)
Waking unrefreshed	2.61 (±0.57)	2.75 (±0.47)	1.62 (1.40–1.88)
Cognitive dysfunction	2.30 (±0.69)	2.45 (±0.62)	1.38 (1.24–1.54)
Fibromyalgia Severity (FS)—(WPI score plus SSS)	19.74 (±3.80)	21.21 (±3.96)	1.10 (1.07–1.13)
Pain intensity (7 days)	7.59 (±1.60)	8.29 (±1.45)	1.32 (1.24–1.40)
Pain interference (activities)	7.38 (±2.27)	8.59 (±1.52)	1.41 (1.32–1.50)
Tampa Scale for Kinesiophobia (TSK)	43.96 (±8.32)	47.99 (±7.48)	1.06 (1.05–1.07)
Central Sensitization Inventory (CSI)	67.17 (±11.86)	70.69 (±11.26)	1.02 (1.01–1.03)
Pain Catastrophizing Scale (PCS)—total score	34.98 (±10.51)	39.15 (±9.59)	1.04 (1.03–1.05)
PCS—Magnification	7.86 (±3.01)	8.84 (±2.75)	1.12 (1.08–1.16)
PCS—Helplessness	15.40 (±5.04)	17.66 (±4.59)	1.09 (1.07–1.12)
PCS—Rumination	11.72 (±3.20)	12.64 (±2.94)	1.08 (1.05–1.11)
Fibromyalgia Impact Questionnaire (FIQ)	67.71 (±12.98)	75.99 (±11.51)	1.06 (1.05–1.07)
Patient Health Questionnaire-9 (PHQ-9)	16.07 (±6.09)	18.68 (±5.77)	1.08 (1.06–1.09)
Duration of fibromyalgia (yrs.)	15.41 (±11.31)	14.98 (±11.86)	0.99 (0.98–1.01)
Age at onset of fibromyalgia (yrs.)	34.25 (±12.03)	29.89 (±11.09)	0.96 (0.95–0.97)
Pain-related comorbidities *n* (%)			
Coexisting nociceptive musculoskeletal pain (yes)	427 (40.8)	619 (59.2)	2.0 (1.68–2.38)
Coexisting neuropathic pain (yes)	292 (42.7)	392 (57.3)	1.78 (1.48–2.14)
Coexisting nociceptive + neuropathic pain (yes)	163 (39.6)	249 (60.4)	2.12 (1.65–2.73)
Physical activity (yes)	532 (50.3)	526 (49.7)	0.98 (0.83–1.15)
History of psychiatric diagnosis *n* (%)			
Psychiatric diagnosis (yes)	457 (44.5)	570 (55.5)	1.55 (1.32–1.83)
Major depression (yes)	363 (42.3)	496 (57.7)	1.74 (1.45–2.08)
Anxiety disorder (yes)	372 (45.2)	451 (54.8)	1.48 (1.23–1.77)
Bipolar disorder (yes)	80 (38.5)	128 (61.5)	1.65 (1.19–2.29)
Panic disorder (yes)	86 (37.1)	146 (62.9)	1.94 (1.42–2.66)
Post-traumatic stress disorder (PTSD) (yes)	48 (34.0)	93 (66.0)	2.05 (1.39–3.02)
Number psychiatric diagnoses	0.92 (±1.20)	1.24 (±1.37)	1.18 (1.10–1.26)
Life adverse events *n* (%)			
Persistent physical aggression (yes)	206 (41.4)	292 (58.6)	1.36 (1.09–1.69)
Persistent verbal aggression (yes)	358 (49.0)	372 (51.0)	1.08 (0.90–1.29)
Persistent emotional aggression (yes)	522 (48.5)	554 (51.5)	1.06 (0.90–1.25)
Sexual abuse (yes)	163 (44.7)	202 (55.3)	1.28 (1.00–1.63)
Cumulative number of abuse events	1.51 (±1.39)	1.60 (±1.45)	1.05 (1.00–1.10)
Onset after major event (yes)	522 (47.8)	570 (52.2)	1.18 (1.00–1.39)

Data were presented as mean (SD) or (%), odds ratio (OR) with confidence interval (CI 95%).

**Table 3:** Continuous variables are presented as mean (SD), and categorical variables as *n* (%). Odds ratios (OR) with 95% confidence intervals are shown at the step in which each variable entered the hierarchical model.

Variable	Block	No Disability	Disability	OR (95% CI)	*p*
Age (years)	1	50.10 (±10.99)	51.28 (±9.40)	1.01 (1.00–1.02)	<0.001
Smoking status	1	138 (13.3%)	183 (17.3%)	1.40 (1.10–1.79)	0.007
Alcohol use	1	640 (61.8%)	504 (47.5%)	0.56 (0.47–0.67)	<0.001
Pain-related comorbidities	2	1.60 (±0.75)	1.84 (±0.78)	1.39 (1.24–1.57)	<0.001
Fatigue	2	2.55 (±0.55)	2.76 (±0.45)	1.79 (1.48–2.18)	<0.001
Sleep disturbance (non-restorative sleep)	2	2.61 (±0.57)	2.75 (±0.47)	1.27 (1.05–1.53)	0.013
Systemic biopsychosocial burden index	3	−0.10 (0.61)	0.10 (0.61)	1.28 (1.08–1.51)	0.004
Pain severity index	4	−0.22 (0.76)	0.21 (0.66)	1.96 (1.70–2.27)	<0.001
Psychocognitive burden index	5	−0.18 (0.78)	0.17 (0.70)	1.35 (1.15–1.58)	<0.001
Psychopharmacological load index	6	1.30 (0.91)	1.64 (0.96)	1.25 (1.12–1.39)	<0.001
Analgesic load index	6	−0.12 (0.76)	0.12 (0.80)	1.10 (0.97–1.25)	0.140
Tampa Scale for Kinesiophobia	7	43.96 (±8.32)	47.99 (±7.48)	1.03 (1.02–1.05)	<0.001

Block 1—Sociodemographic factors: Age (years), Smoking status, Alcohol use (*Alcohol Use Disorders Identification Test Consume—AUDIT-C*). Block 2—Clinical pain phenotype: Pain-related comorbidities, Fatigue, Sleep disturbance (non-restorative sleep). Block 3—Systemic burden. Composite index representing biopsychosocial systemic burden: mean [z (cumulative abuse), z (central sensitization), z (number of chronic diseases)]. Block 4—Pain severity. Composite index of pain burden: = mean [z (*Widespread pain index—WPI*), z (pain intensity in the last 7 days), z (pain interference with activities)]. Block 5—Psychocognitive burden. Composite index of affective-cognitive burden: mean [z (*Patient Health Questionnaire-9—PHQ-9*), z (*Pain Catastrophizing Scale—PCS total*), z (cognitive symptoms)]. Block 6—Medication burden. Medication-related indices: sum of anticonvulsants, any antidepressant, benzodiazepines, and zolpidem; analgesic load index = mean [z (number of non-opioid analgesics), z (number of opioid analgesics)]. Block 7—Kinesiophobia (proximal): *Tampa Scale for Kinesiophobia*.

## Data Availability

The datasets generated and/or analyzed during the current study are available from the corresponding author upon reasonable request. All data are fully de-identified to protect participant confidentiality. Requests for data access can be directed to wcaumo@hcpa.edu.br.

## Abbreviations

ACR	American College of Rheumatology
AUDIT-C	Alcohol Use Disorders Identification Test—Consumption
BMI	Body Mass Index
COPD	Chronic Obstructive Pulmonary Disease
CSI	Central Sensitization Inventory
FIQ	Fibromyalgia Impact Questionnaire
FM	Fibromyalgia
FS	Fibromyalgia Severity (WPI + SSS)
HCPA	Hospital de Clínicas de Porto Alegre
ICF	Informed Consent Form
IRB	Institutional Review Board
NPS	Numeric Pain Scale
NSAIDs	Non-Steroidal Anti-Inflammatory Drugs
PCS	Pain Catastrophizing Scale
PHQ-9	Patient Health Questionnaire-9
PTSD	Post-Traumatic Stress Disorder
REDCap	Research Electronic Data Capture
SMD	Standardized Mean Difference
SOC	Sense of Coherence
SSRI	Selective Serotonin Reuptake Inhibitor
SSS	Symptom Severity Scale
STROBE	Strengthening the Reporting of Observational Studies in Epidemiology
TSK	Tampa Scale for Kinesiophobia
UFRGS	Universidade Federal do Rio Grande do Sul
WPI	Widespread Pain Index
